# Indigenous use of fire in the paramo ecosystem of southern Ecuador: a case study using remote sensing methods and ancestral knowledge of the Kichwa Saraguro people

**DOI:** 10.1186/s42408-022-00164-1

**Published:** 2023-01-23

**Authors:** Sandy Celi Díaz, Liliana Correa Quezada, Leticia Jiménez Álvarez, Julia Loján-Córdova, Vinicio Carrión-Paladines

**Affiliations:** 1 Carrera de Gestión Ambiental, Universidad Técnica Particular de Loja, San Cayetano Alto s/n, 1101608 Loja, Ecuador; 2 Departamento de Ciencias Jurídicas, Universidad Técnica Particular de Loja, San Cayetano Alto s/n, 1101608 Loja, Ecuador; 3 Departamento de Ciencias Biológicas y Agropecuarias, Universidad Técnica Particular de Loja, San Cayetano Alto s/n, 1101608 Loja, Ecuador

**Keywords:** Kichwa Saraguro, Traditional use of fire, Wildfires

## Abstract

**Background:**

The Indigenous Kichwa Saraguro people of southern Ecuador have long relied on traditional burning to manage their environment. However, their traditional use of fire in one of the most important ecosystems in southern Ecuador, the herbaceous paramo, is not well known. This lack of knowledge does not allow for the improvement of local regulations related to integrated fire management, which is a shortcoming compared to other regulations applied in South America. In this context, and to understand the impacts of the Indigenous use of fire, a climatic analysis of the area was carried out, generating a historical climograph (period: years 1981–2021) and four annual climographs that were contrasted with a remote sensing study of fire severity over 4 years (years 2018, 2019, 2020, and 2021). In addition, traditional fire use was determined through the application of semi-structured interview questionnaires applied to 61 women and 89 men, whose data were analyzed with the level of information fidelity (LIF), informant consensus factor (ICF), and principal component analysis (PCA). Therefore, in this study, we argue that it is important to incorporate the concepts of (i) wildfire severity and (ii) cultural burning in wildfire policies and regulations in southern Ecuador.

**Results:**

The results indicate that low-severity fires occur within the Saraguro territory and that fire use knowledge is transmitted to new generations incorporating both how and where to perform traditional burning. They also know when to burn using the burning calendar that is generally applied during the climatic phenomenon known as “Veranillo del Niño” (VdN).

**Conclusions:**

These results can help decision-makers design policies, regulations, and proposals for the correct use of fire as a tool for the management of ecosystems in southern Ecuador affected by wildfires. In addition, the results can be used to improve the National Strategy for Integrated Fire Management 2021–2025 promoted by the Ministry of Environment, Water and Ecological Transition of Ecuador.

## Resumen

**Antecedentes:**

El pueblo indígena de los Kichwa-Saraguro del sur de Ecuador ha confiado en las quemas tradicionales para manejar su ambiente. Sin embargo, su uso tradicional en uno de los ecosistemas más importantes en el sur de Ecuador, el páramo herbáceo, no es bien conocido. Esta falta de conocimiento no permite el mejoramiento de las regulaciones locales relacionadas con el manejo integrado del fuego, lo cual significa una anormalidad comparada con otras regulaciones aplicadas en América del Sur. En este contexto y para entender los impactos del uso indígena del fuego, un análisis climático del área fue llevado a cabo, generando un climograma histórico (desde 1981 a 2021) y cuatro climogramas anuales que fueron contrastados con un estudio de severidad del fuego mediante sensores remotos por cuatro años (2018, 2019, 2020 y 2021). Adicionalmente, el uso tradicional del fuego fue determinado mediante la aplicación de cuestionarios semiestructurados a 61 mujeres y 89 hombres, y cuyos datos fueron analizados mediante la fidelidad del nivel de información (LIF), el factor de consenso del informante (ICF) y un análisis de componentes principales (PCA). Por lo tanto, argüimos que es importante incorporar los conceptos de i) severidad de los incendios y ii) quemas culturales, en las políticas y regulaciones del manejo de fuego en el sur de Ecuador.

**Resultados:**

Los resultados indican que los fuegos de baja severidad ocurren dentro del territorio del Saguaro y que el conocimiento sobre el uso del fuego es transmitido a nuevas generaciones incorporando tanto el cómo y el cuándo hacer las quemas tradicionales. Ellos saben también cuándo quemar mediante el uso del calendario de quemas, que es generalmente aplicado durante el fenómeno climático conocido como Veranillo del Niño (VdN).

**Conclusiones:**

Estos Resultados pueden ayudar a los decisores a diseñar políticas, regulaciones, y propuesta para el uso del fuego como herramienta de manejo de los ecosistemas del sur de Ecuador afectados por incendios. Adicionalmente, los resultados pueden usarse para mejorar la Estrategia Nacional para el Manejo Integrado del Fuego 2021-2025 promovido por el Ministerio de Ambiente, Agua y Transición Ecológica de Ecuador.

## Background

The use of remote sensing methods through the analysis of satellite images is a transcendental field for wildfire research that has been widely explored in the last decade (White 2019; Barboza Castillo et al. 2020). Remote sensing has made it possible to characterize the wildfire regime by severity levels in several countries around the world (e.g., Delegido et al. 2018; Santos et al. 2020). However, few such studies exist in Ecuador, but include the work of Reyes-Bueno and Loján-Córdova (2022) who evaluated three techniques for monitoring wildfire susceptibility, and the work of Cabrera et al. (2020) who determined a reproducible methodology for wildfire identification and estimation of vegetation recovery. In this context, further research is needed in Ecuador to determine the severity of wildfires, not only using remote sensing methods but also including the ancestral knowledge of fire use by the 14 Indigenous nationalities that inhabit the territory (Trujillo and Poveda 2012; Cayo-Vega 2022). In this way, it will be possible to obtain more data that will contribute to the integrated management of wildfires (Paudel 2021).

The inclusion of human knowledge of fire use in remote sensing studies of wildfires has gained much interest in recent decades (Dennis et al. 2005). This is because human use of fire in ecosystems has ancestral origins and can be traced back to Indigenous peoples in Latin America, North America, Africa, Asia, Europe, and Oceania (e.g., Butz 2009; Pyne 2012; Fache and Moizo 2015; Long et al. 2021). In these regions, where these practices continue, they provide important benefits to ecosystems and Indigenous communities as demonstrated by recent research (Hankins 2015; Trauernicht et al. 2015; Halpern 2016; Bird et al. 2018; Lake and Christianson 2020; Hart-Fredeluces et al. 2021; Marks-Block et al. 2021; Adlam et al. 2021). However, the criminalization of traditional fire management has eroded the ability of Indigenous peoples and individuals to put their knowledge into practice (Lewis et al. 2018; Norgaard and Tripp 2019). As a result, ecosystems that depend on cultural fire regimes have deteriorated and traditional livelihoods that depend on these ecosystems have been threatened (Long et al. 2017). Thus, Williams (2013) determined that fire exclusion policies are causing fuels to accumulate in ecosystems that are generally susceptible to wildfire. This, combined with climate change, produces greater threats to both human communities and ecosystems (Pausas and Keeley 2021). Therefore, decision-makers are increasingly interested in supporting the efforts of Indigenous nations to revitalize their knowledge of cultural burning practices (e.g., Marks-Block and Tripp 2021).

In the Americas, the use of fire in agriculture is known as the slash-and-burn system, which consists of felling large trees with an ax and cutting shrubs, grasses, and vines with a machete (Rojas Rabiela 1982). This system allows the freshly cut biomass to be piled and then burned and upon the top layer of ash resulting from the burning, traditional farmers plant crops such as maize (*Zea mays* L.) and beans (*Phaseolus vulgaris* L.) for local food (Ponce et al. 2012). In addition, other frequent and low-intensity burning techniques are carried out, which correspond to so-called cultural or traditional burning (Long et al. 2021). In many cases, cultural burning is used as a natural resource management tool. For example, Anderson (1996) reported that, in California, at least twenty tribes conduct small-scale burning to protect and stimulate the production of reeds (*Muhlenbergia rigens*: Poaceae) and to prohibit shrub or tree encroachment. Likewise, Long et al. (2021) determined that these Indigenous practices improve the edaphic properties of the soils and serve to increase food, medicine, and fiber production. Forests are also burned to provide staple foods for Indigenous peoples (Bowcutt 2013) and various types of ecosystems are burned to harvest wild animals and insects (Anderson 1996; Long and Lake 2018). Finally, many tribes use cultural burning to stimulate the production of berry-producing shrubs such as blueberries (*Vaccinium* spp.); the production of geophytes (root crops or edible Indian potatoes) that propagate from subway storage organs; and the production of seed-producing grasses and herbs (Anderson 1997; Anderson and Rowney 1999).

These practices were applied during the conquest and the colonial times, although the conquerors always tried to modify them (Taylor et al. 2016). For example, more than a century ago, some land managers denounced Indigenous burning, and later some derided it as “Paiute forestry,” being detrimental to the forests (Greeley 2000). However, historical records and current studies show that the traditional burning by many aboriginal groups is still preserved and continues to operate in many Latin American ecosystems (Messina and Cochrane 2007) and serves as a territorial management tool. Such is the case of the Tarahumara or Rramuri in Mexico, who consider that burning attracts rainfall and also eliminates weeds from agricultural fields, stimulating the growth of pastures (Fulé et al. 2011). Similarly, the Pemon of Venezuela use fire to prevent large fires, to communicate with each other, for the clearing of fishing and hunting trails, to rejuvenate savanna grasslands for wild animals or livestock, and to prepare the soil for crops (shifting agriculture) (Rodríguez 2007). In the Amazon, the Xavante ethnic group (central Brazil) is especially known for burning vegetation associated with ceremonial events and during the hunting of large groups of mammals (de Melo and Saito 2013; Welch and Coimbra Jr. 2021). However, it remains to be clarified whether such traditional fires are of low severity (Keeley 2009) and whether indigenous people employ this type of burning knowing that they are part of the natural dynamics of ecosystems.

In Ecuador, some Indigenous groups continue to maintain the traditional practice of slash-and-burn and cultural burning as tools for the establishment of new cultivation areas. This is corroborated by some studies that have demonstrated the use of these ancestral practices in the ecosystems of the Amazon basin of northeastern Ecuador (Messina and Cochrane 2007; Schritt et al. 2020). In addition, a particularly important ecosystem that is burned in Ecuador is the paramo. This ecosystem serves water regulation functions due to precipitation that tends to be of high frequency and low intensity (Buytaert et al. 2006; Echeverry and Leiton 2020). This ecosystem maintains horizontal precipitation, i.e., precipitation due to fog and dew, which can add an unknown amount of water, as occurs with hidden precipitation in montane cloud forest, where it usually adds 5–20% of that of ordinary precipitation (Ataroff and Rada 2000). Due to its wide distribution in the Andes, paramo thrives in a high variability of environmental conditions (Jiménez-Rivillas et al. 2018). These variations are due to the geographical position of each one, so they also present climatic variations (Bendix et al. 2013) and extreme daily variations in ambient temperature, light intensity, relative humidity, and multiannual precipitation (Van der Hammen et al. 2007). In this context, one of the groups that make use of the paramos of southern Ecuador is the indigenous Saraguro (Pohle 2008). According to Armijos et al. (2016), the population of the Saraguro ethnic group is about 60,000 people. They form one of the most organized communities among the Indigenous nationalities of Ecuador and have one of the most important biodiversity niches in South America (e.g., Podocarpus National Park, home to some 4,000 species of vascular plants) (Jørgensen and León-Yánez 1999; Valencia 2000). This Indigenous group is constantly threatened by globalization which produces a series of cultural, economic, and political changes that are usually considered as an increase in interdependence, integration, and interaction between people and organizations around the world (Lauderdale 2008). The main people affected are young people who are losing their dialect, customs, and traditional beliefs. However, there are many cases where traditional knowledge is maintained, especially in the use of medicinal plants, where the uses of these plants have been investigated for the isolation of metabolites for applications in the pharmaceutical and cosmetic industries (Armijos et al. 2016). Regarding the use of fire, no studies have yet been in Saraguro to determine the causes and timing of burning in the paramo, one of the most important ecosystems of southern Ecuador. In addition, it remains to be clarified whether these traditional fires are of high, medium, or low severity (Keeley 2009) and whether the indigenous people of Saraguro use this type of burning knowing that they are part of the natural dynamics of the ecosystems. In this context, for the Saraguro, the use of *wakanina* (“sacred fire” in Kichwa) and *nina manalli* (“harmful fire” in Kichwa) has been passed down from generation to generation to manage their natural resources, but it is not clear if these indigenous people can determine the effects of these types of fires that can positively or negatively affect *pachamama* (nature in Kichwa). Therefore, it is necessary to identify the Indigenous use of fire by the Saraguro people to meet the requirements for the formulation of an integral program for the sustainable management of natural resources. Furthermore, in Ecuador, some laws do not include the concept of fire severity and the ancestral knowledge of native peoples regarding the use of fire as it occurs in other regions of the world (e.g., Stephens and Ruth 2005) and yet punish with imprisonment those who carry out agricultural burning that becomes uncontrollable (PENAL-COIP, Código Orgánico Integral 2014).

The objective of this study was to determine the Indigenous use of fire by the Saraguro people in the paramo ecosystem of southern Ecuador. To understand the environmental context of the study area, climographs were generated with NASA meteorological data and fire severity maps at the landscape level compared with fire severity in the paramo ecosystem over the last 4 years (2018, 2019, 2020, and 2021). Ancestral knowledge on the use of fire was obtained with permission of the Indigenous people of Saraguro, applying semi-structured interviews and participatory workshops. The connection between the remote sensing method and local knowledge allowed us to understand the degree of severity of fires and the reasons for the Indigenous use of fire. Decision-makers can use these tools and results to generate integrated wildfire management standards in this type of cultural landscapes.

## Materials and methods

### Study area

**Fig. 1:**
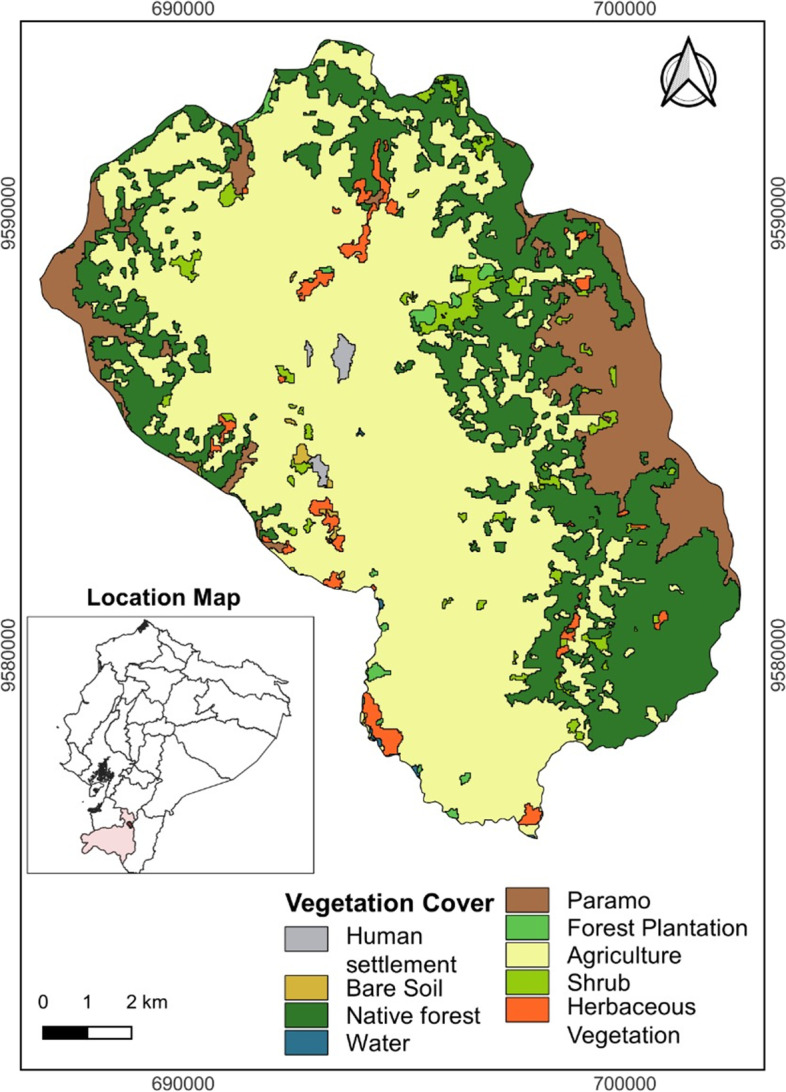
Location of the study area, canton of Loja, San Lucas parish, southern Ecuador

The research was conducted in the province of Loja, canton Loja, in the Indigenous parish of San Lucas in southern Ecuador (3° 44′ 47.64″ south and 79° 15′ 58.45″ west) (Fig. 1). The ecosystem is classified as humid montane forest (HMF) with the presence of herbaceous paramo in the higher elevations (Holdridge 1979; Sierra et al. 1999). It is located at an elevation between 2525 and 3010 m a.s.l with irregular topography (Andrade et al. 2017). The study area has a temperate climate, with temperatures ranging between 12 and 18 °C throughout the year and rainfall ranging between 600 and 1000 mm per year (SAN LUCAS GAD PARROQUIAL; LOJA, Cantón 2015). The land tenure of the paramos corresponds to the legal figure recognized as ancestral “possession of community use” (in this case of the Saraguro people), which is protected by the current Organic Law of Rural Lands and Ancestral Territories (Ley Orgánica de Tierras Rurales y Territorios Ancestrales 2018), which applies throughout Ecuador. In addition, these paramos are part of the buffer zone of the Corazón de Oro Protected Forest, which is protected by the community-indigenous management vision of the parish councils (in this case, the parish council of San Lucas—Saraguros). This Community-Indigenous management is protected by the Forestry and Conservation of Natural Areas and Wildlife Law, which allows for the protection of forests and protective vegetation in private and community areas in Ecuador (Ley Forestal y de Conservación de Áreas Naturales y Vida Silvestre 2004). However, this protection model differs from Ecuador’s national system of protected areas, the SNAP, which is managed by the Ecuadorian state (Ley para la Preservación de Zonas de Reserva y Parques Nacionales, 2009).

The predominant economic activities are agriculture, livestock, and forestry (Coronel-Alulima 2019). Agricultural production is characterized by the cultivation of corn (*Zea mays*), beans (*Phaseolus vulgaris*), broad beans (*Vicia faba* L.), potatoes (*Solanum tuberosum* L.), peas (*Pisum sativum* L.), peaches (*Prunus persica* L.), apples (*Malus domestica* L. Borkh), and medicinal plants and flowers that are intended for self-consumption (SAN LUCAS GAD PARROQUIAL; LOJA, Cantón 2015). However, San Lucas also has livestock production which is an environmental problem, due to the expansion of the agricultural frontier, which causes changes in vegetation cover (SAN LUCAS GAD PARROQUIAL; LOJA, Cantón 2015; Coronel-Alulima 2019). In addition, the Saraguro Indigenous people carry out the traditional burning of paramo vegetation, which leads to renewing pastures that serve as fodder, especially for cattle (*Bos taurus*) (Aguirre 2017).

### Determination of fire weather and severity of wildfires

**Fig. 2:**
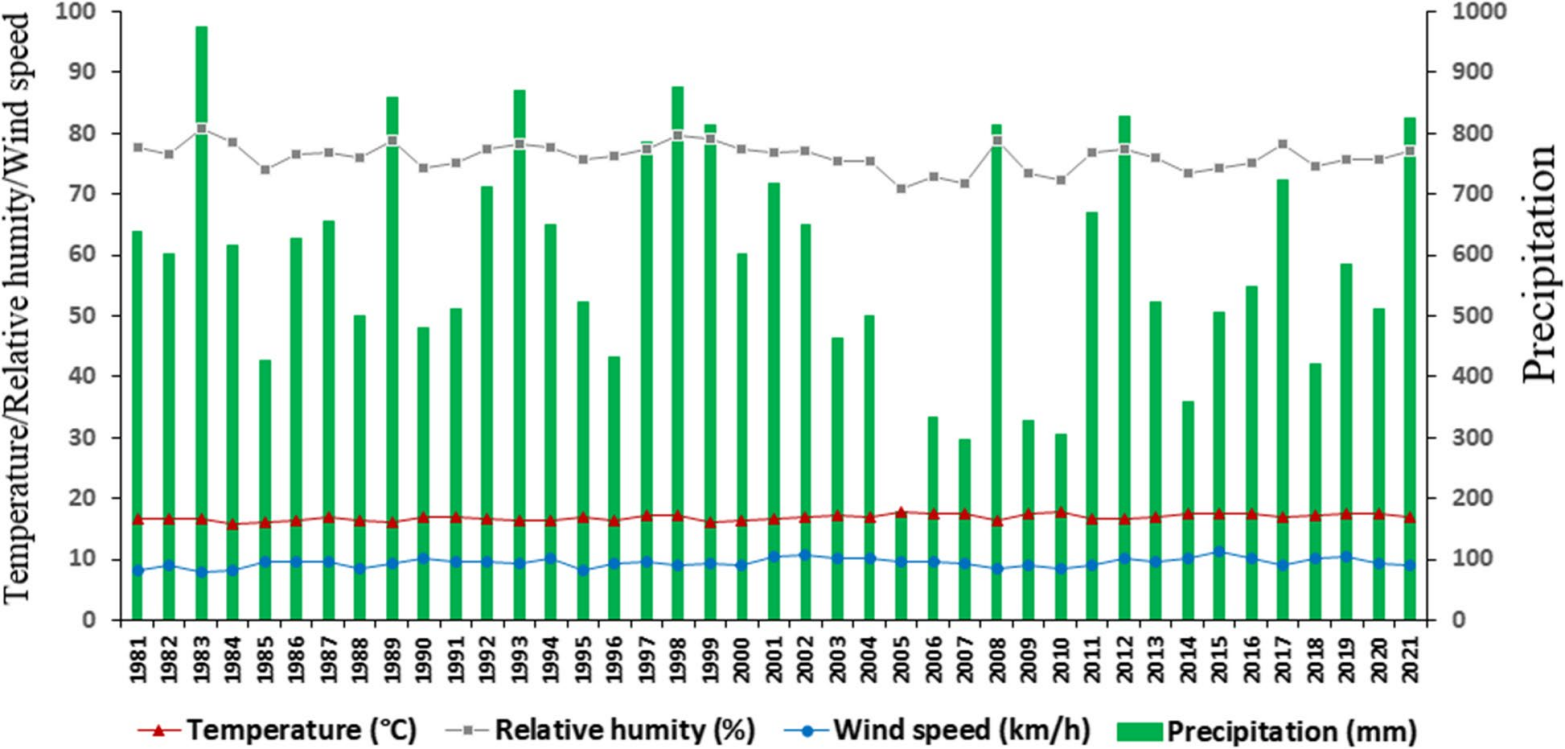
Interannual climograph of the paramo zone of southern Ecuador, generated with NASA meteorological data (period: 1981–2021)

**Fig. 3:**
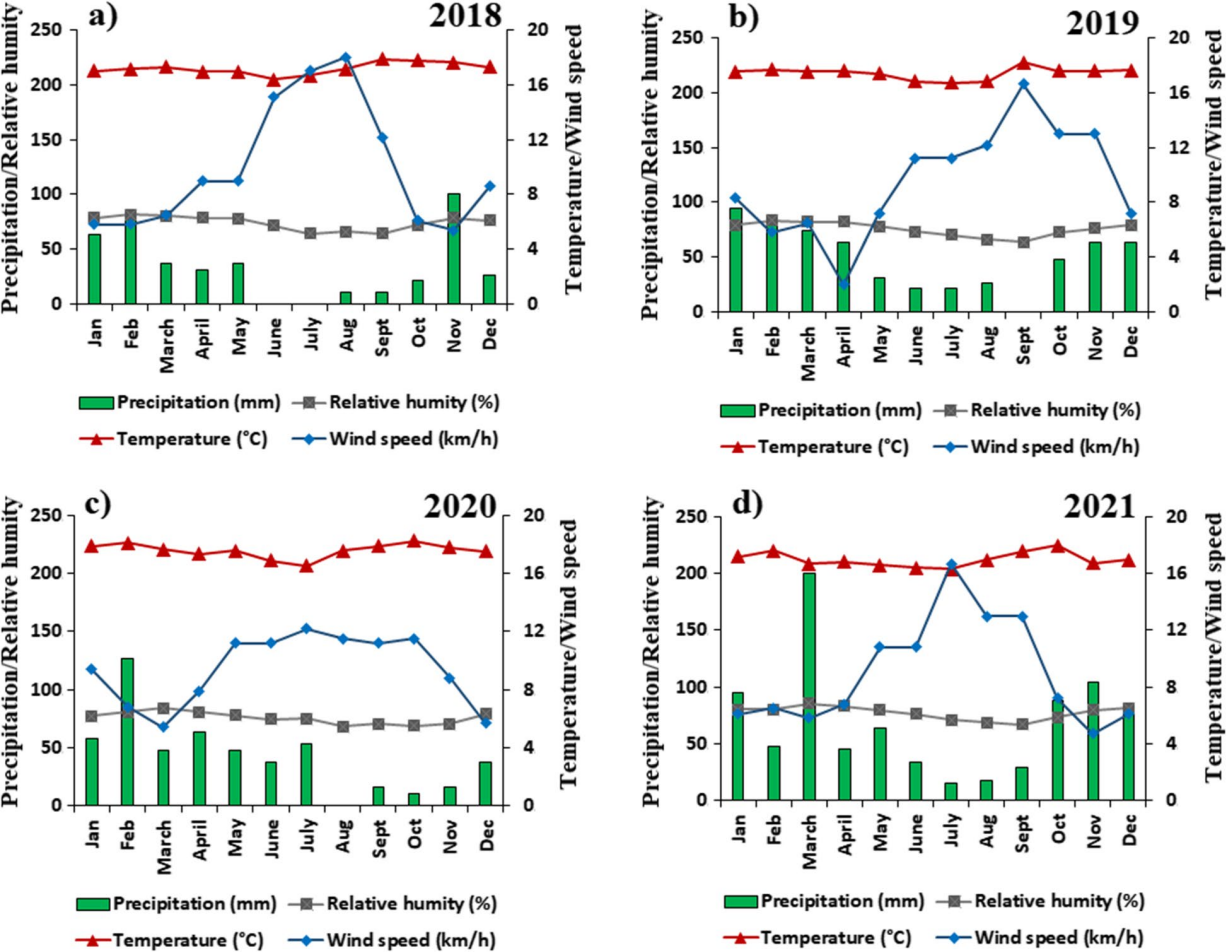
Climographs of the paramo zone of southern Ecuador, generated with NASA weather data. **a** Corresponds to the year 2018. **b** Corresponds to the year 2019. **c** Corresponds to the year 2020. **d** Corresponds to the year 2021

The fire weather was determined using NASA meteorological data (https://power.larc.nasa.gov/data-access-viewer/ accessed on February 5, 2022) corresponding to temperature (°C), precipitation (mm), relative humidity (%), and wind speed (m/s converted to km/h) (Rodrigues and Braga 2021; Carrión-Paladines et al. 2022). The meteorological data were generated considering the paramo zone whose geographical coordinates are −3.727639° and −79.205111°. With the data, the historical climograph considering the last 41 years (period 1981–2021) and the respective annual climographs for the last 4 years (2018, 2019, 2020, and 2021) were generated (Figs. 2 and 3). In addition, with the meteorological data of the last 4 years, the monthly average for precipitation (mm), relative humidity (%), temperature (°C), and wind speed (km/h) were calculated in order to determine the two climatic phases known as the rainy period and annual drought period (Rollenbeck and Bendix 2011a, 2011b). Both the historical climograph and the annual climographs were used for the interpretation of traditional fire use and fire severity indices.

The evaluation of the severity of wildfires was carried out at two levels. The first was the calculation of severity considering the entire San Lucas parish and the other considering the paramo ecosystem, to contrast the effect of the fires between the different land uses related to the paramo vegetation and to be able to contextualize with a more regional vision. The severity was calculated using images from the Sentinel 2B satellite (the years 2018, 2019, 2020, and 2021) with its MSI multispectral sensor. The dates with the lowest cloud cover (<20% cloud cover) of the last months of the year were considered, which is where the highest incidence of wildfires occurs (Řezník et al. 2021). We used the normalized burned area index (NBR) designed to highlight burned areas by their spectral signature (van Dijk et al. 2021). For this analysis, we used the following formula (Parker et al. 2015).

$$\textrm{NBR}=\frac{\left({R}_{\textrm{NIR}}-{R}_{\textrm{SWIR}}\right)}{\left({R}_{\textrm{NIR}}+{R}_{\textrm{SWIR}}\right)}$$

$${R}_{\textrm{NIR}}=\textrm{reflectivity}\ \textrm{in}\ \textrm{the}\ \textrm{band}\ \textrm{NIR}\ \left(\textrm{B}8\textrm{A}\right)$$

$${R}_{\textrm{SWIR}}=\textrm{reflectivity}\ \textrm{in}\ \textrm{the}\ \textrm{band}\ \textrm{SWIR}\ (B12)$$

**Fig. 4:**
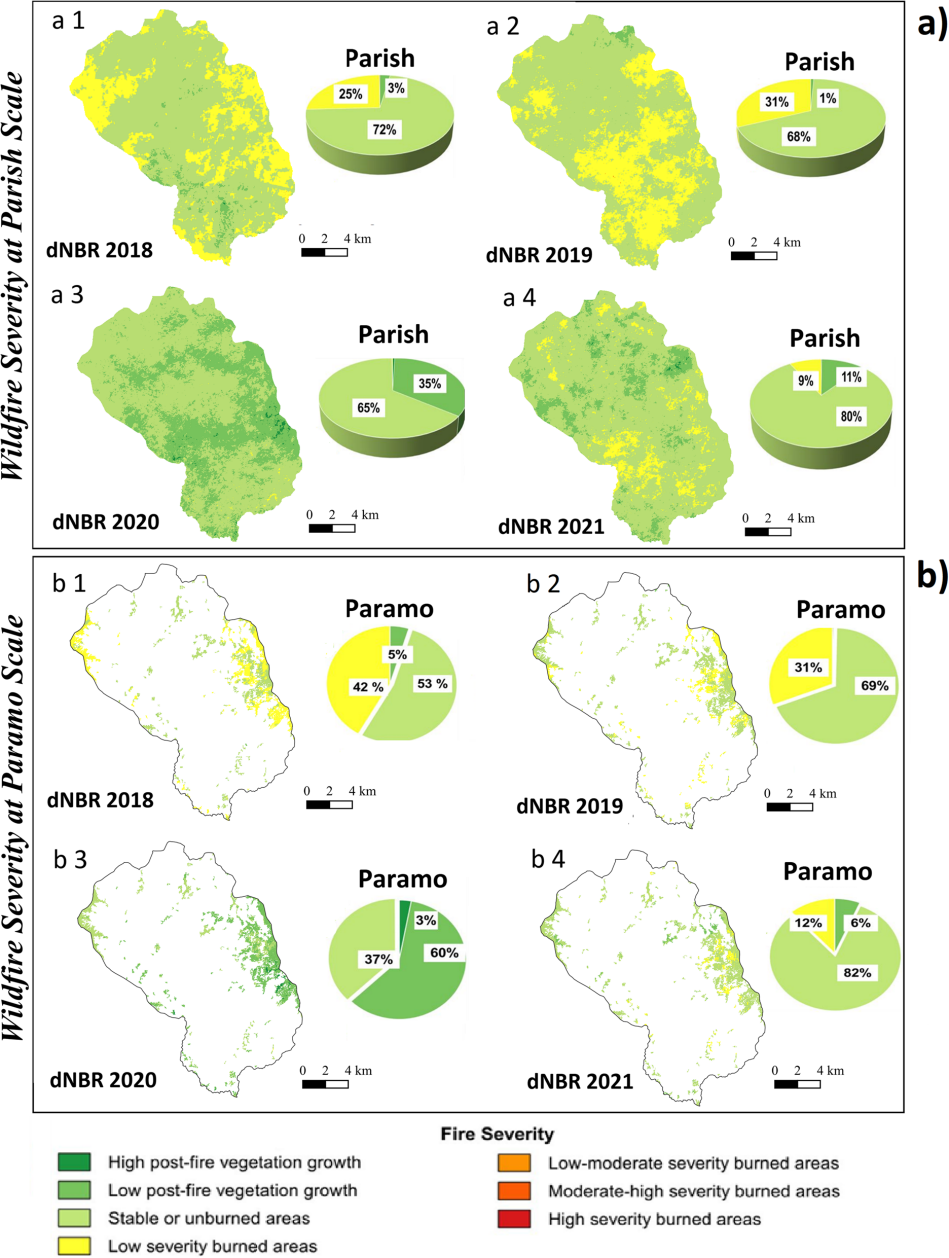
Wildfire severity maps. **a** Wildfire severity at the San Lucas Indigenous parish scale: (a 1) severity for the year 2018; (a 2) severity for the year 2019; (a 3) severity for the year 2020; (a 4) severity for the year 2021. **b** Wildfire severity at the paramo scale: (b 1) severity for the year 2018; (b 2) severity for the year 2019; (b 3) severity for the year 2020; (b 4) severity for the year 2021. The percentage of fire severity is presented for each contrasting year

Likewise, dNBR, the difference between pre-fire and post-fire (NBR Prefire − NBR Postfire), was calculated to estimate the severity using the respective formula (Santos et al. 2020) (Fig. 4).

$$\textrm{dNBR}=\left({\textrm{NBR}}_1-{\textrm{NBR}}_2\right)$$

$${\textrm{NBR}}_1=\textrm{pre}-\textrm{fire}\ \textrm{burned}\ \textrm{area}\ \textrm{index}$$

$${\textrm{NBR}}_2=\textrm{post}-\textrm{fire}\ \textrm{burned}\ \textrm{area}\ \textrm{index}$$

### Collection of data on indigenous use of fire in the paramo of southern Ecuador

Data were obtained through a semi-structured questionnaire applied to 61 women and 89 men (150 in total) of the Kichwa Saraguro people containing closed and open questions. The ages of the informants were from 20 to 60 and > 60 years. The questionnaires included questions related to general information on the characteristics of the producers and their agricultural activities, the use of fire in the paramo ecosystem, ancestral knowledge about the technique used to carry out the burns, the effects, and the training received on this topic. In addition, participatory workshops were held with key people (community leaders) to determine the appropriate months to perform the burns, and learn about techniques—ignition patterns, and a burn calendar was developed for the study area (McKemey et al. 2020a, 2020b).

### Quantitative data analysis

The historical uses and the effects of fire in the paramo were evaluated by calculating the level of information fidelity (LIF) and the informant’s consensus factor (ICF). The LIF was calculated using the following formula, as cited in the literature (Yaseen et al. 2015):

$$\textrm{LIF}=\textrm{Ip}/\textrm{Iu}\times 100$$

where:

Ip is the number of informants that indicate a characteristic related to the use of fire, and Iu is the number of informants that indicate all the characteristics related to the use of fire. The high value of the LIF confirms that a characteristic of the use of fire is used a lot, while the low value of the LIF confirms a low frequency of the characteristic of fire use. The LIF reflects the degree of preference of a particular fire use characteristic over others.

The ICF was used to measure the agreement or consensus among all informants (among all respondents) on each fire use characteristic. In this study, the ICF was calculated using the following formula (Khan et al. 2015):

$$\textrm{IFC}=\left(\textrm{Nt}-\textrm{Nur}\right)/\left(\textrm{Nt}-1\right)$$

where:

Nt is the number of informants surveyed and Nur is the number of informants who indicated the main use they are making with fire. The ICF results vary from 0 to 1, where low values (close to 0) show that a characteristic of the use of fire is common and there is an exchange of information (or there is consensus among the informants). When high values are obtained (close to 1), they show that a characteristic of the use of fire is carried out at random or there is no exchange of information (there is no consensus) about its use among the informants. In addition, with the data from the surveys, the answers given by women and men were considered, so an analysis of knowledge by gender about the use of fire was carried out, as carried out by Bhagawati et al. (2017).

### Legal remedies in Ecuadorian wildfire

**Fig. 5:**
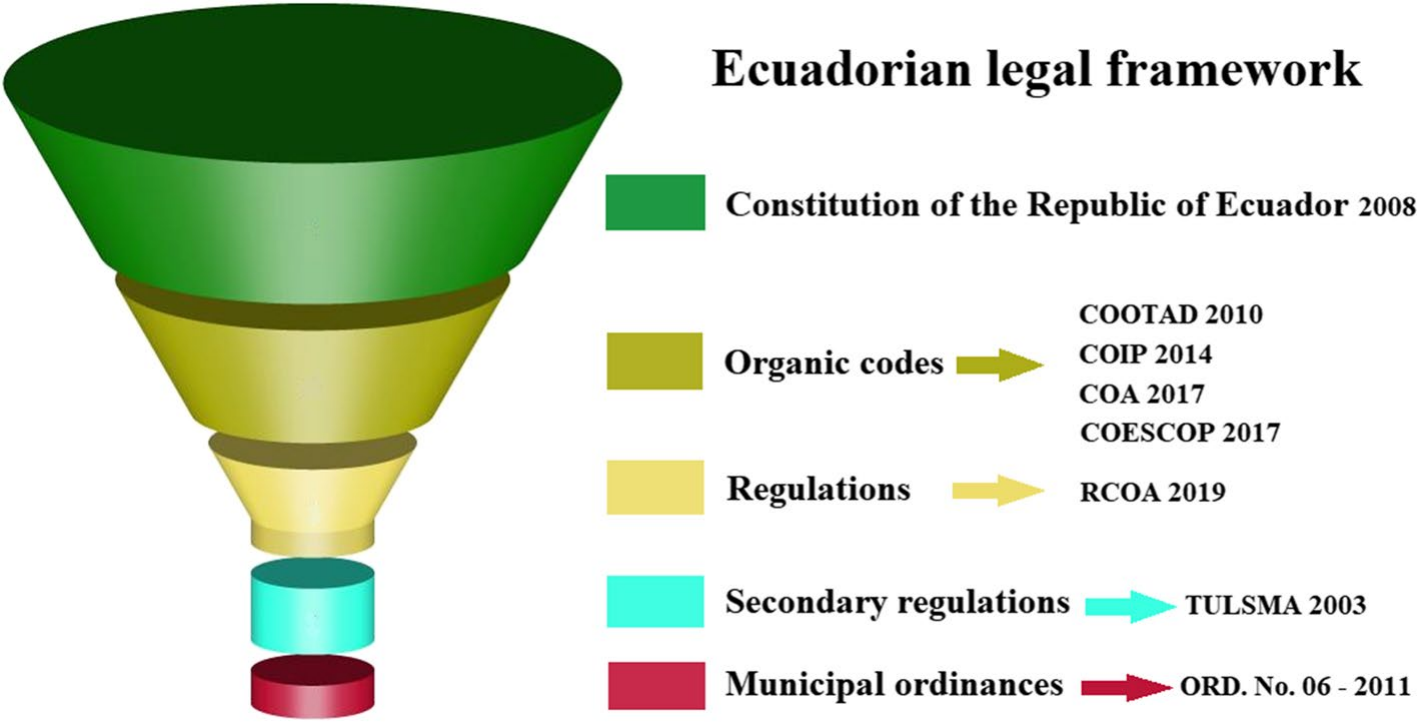
Legal framework for integrated fire management (IMF) in Ecuador

Although we can now determine the severity of the fires, there is still a legal vacuum in Ecuadorian legislation that does not reflect the different types of severity in terms of sanctions (Fig. 4). Therefore, we conducted a legal analysis of Ecuadorian legislation and compared it with that of other countries to determine whether environmental infractions could exist within the study area and to suggest certain reforms, more in line with the reality of the Indigenous people. In this context, Fig. 5 represents the Ecuadorian legal framework in the form of a funnel. This shape allows for channeling all laws, norms, and ordinances related to wildfires. This channeling and distribution starts with the Constitution of the Republic (general) and ends with the Municipal Ordinances (specific) as shown in Fig. 5.

Figure 5 is described below.

- ✓ The 2008 Constitution of the Republic (general level) establishes the principles of interculturality and protection of nature as a subject of rights, as well as the guidelines to be followed at the institutional and national levels. Article 264 states: To administer the services of prevention, protection, aid, and fire extinction.
- ✓ Article 264 of the 2008 Constitution of the Republic of Ecuador is in accordance with articles 55, 136, 140, 541 of the Organic Code of Territorial Ordering (COOTAD https://www.cpccs.gob.ec/wp-content/uploads/2020/01/cootad.pdf) and 25 and 27 of the Organic Administrative Code (COA, https://www.gobiernoelectronico.gob.ec/wp-content/uploads/2020/11/COA. pdf), where fire prevention, protection, relief, and extinguishing services are declared as the exclusive competence of the Cantonal Decentralized Autonomous Governments (specific level such as municipal ordinances through the fire department). Such is the case in the study area where the Municipality of Loja applies Ordinance No. 06-2011 on forest fires (https://www.loja.gob.ec/files/documentos/2014-10/bomberosloja_1.pdf). This ordinance refers to the planning, control, monitoring, and evaluation of integrated fire management (IFM) proposed at the cantonal level.
- ✓ However, in order for the Municipality of Loja to apply this ordinance (Ordinance No. 06-2011) that safeguards the actions of users of the Saraguro paramo, it must rely on official instruments such as the “National Strategy for Integrated Fire Management (ENMIF)” and the “National Action Plan for ENMIF (PNA-ENMIF),” which is due to art. 377 of the Regulations of the Organic Environmental Code (R-COA, https://site.inpc.gob.ec/pdfs/lotaip2020/REGLAMENTO%20AL%20CODIGO%20ORGANICO%20DEL%20AMBIENTE.pdf).
- ✓ In this context, the Organic Environmental Code (R-COA) regulates the use of fire through art. 384, which states that: “unauthorized burning that triggers a forest fire entails administrative, civil and criminal liability, depending on the damage caused.”
- ✓ Therefore, with respect to criminal sanctions derived from forest fires for the damage caused, art. 246 of the Organic Integral Penal Code (COIP, https://www.defensa.gob.ec/wp-content/uploads/downloads/2021/03/COIP_act_feb-2021.pdf) mentions three types of sanctions:

1. Imprisonment of 1 to 3 years (“except for agricultural or domestic burns carried out by communities or small farmers within their territory”) for those who directly or indirectly provoke or instigate fires in native forests, planted forests, and paramos.
2. Culpable offense with imprisonment of 3 to 6 months for those who provoke uncontrolled burning that generates forest fires
3. A prison sentence of 13 to 16 years if, as a consequence of the fire, the death of one or more persons is caused

There is also another type of secondary regulation for the integral management of fire in Ecuador (considered as support). Among the main instruments are:

- ✓ The Single Text of Secondary Environmental Legislation (TULSMA)
- ✓ Resolutions of the National Emergency and Risk Service of Ecuador (SNGRE)
- ✓ Resolutions of the National Council of Competencies (CNC)

## Results

### Severity of wildfires

Figure 2 shows that in the study area there is an interannual climatology where precipitation is the factor that varies the most. There are wet years (e.g., 1983: 975.5 mm) and dry years (e.g., 2005: 179.3 mm) that can affect the incidence and severity of wildfires. Likewise, Fig. 3 shows the climographs in which it is evident that there are 8 rainiest months (from November to June), in which there is a greater amount of precipitation (494.1 mm average for the sum), greater relative humidity (78.2% above average), and a decrease in wind speed (8.1 km/h on average). As for the 4 driest months, from June to September, they can be considered climatically as the wildfire season. In this window of 4 dry months, there is a marked decrease in precipitation (91.6 mm average for the sum), relative humidity (70.1% on average), increase in temperature (17.4°C on average), and wind speed (12.4 average km/h). The temperature varies minimally, namely 17.3 °C for the rainy months and 17.4 °C for the dry months.

Regarding anthropogenic fires, in general, the greatest number of fires are of low severity throughout the geography of San Lucas parish, producing subsequently, by processes of natural succession, low post-fire population growth (Fig. 4). However, there is marked variability between low-severity fires and low post-fire vegetation growth when compared between contrasting years. Thus, for 2018, the area has 25% low-severity fires with 72% low post-fire vegetation growth. For the year 2019, these percentages change with an increase to 31% of low-severity fires and 68% of low post-fire vegetation growth, while for the year 2020 there were possibly no fires of this type due to the confinement by Covid-19 so 65% corresponded to low post-fire vegetation growth while 35% corresponded to high post-fire vegetation growth. Finally, by 2021, low-severity fires occurred in only 9% of the burned parish territory with 80% of low post-fire vegetation growth. In this context, traditional fire use directly influences the generation of this type of fire, possibly as a function of climatic conditions, fuel content, and human reasons for burning that have been reported in some recent research (e.g., Carrión-Paladines et al. 2022).

On the other hand, anthropogenic fires of low severity also occur in the páramo ecosystem, which is consistent with what occurs at the landscape level (San Lucas parish territory). For example, in 2018, there were 42% low-severity fires with 53% low post-fire vegetation growth, and in 2019, there were 31% low-severity fires with 59% low post-fire vegetation growth, while in 2020 there were no fires due to confinement by Covid-19 but there were 37% corresponding to low post-fire vegetation growth while 60% corresponds to high post-fire vegetation growth and in 2021 there were 12% low-severity fires with 82% low post-fire vegetation growth.

### Traditional use of fire by the Saraguro indigenous people

Table 1 shows the results of the LIF and ICF indices of the information collected for the questions that were affirmative (yes) or negative (no). Almost all respondents from the Saraguro people indicated that their farms have paramos, which shows that there is a consensus among all informants on this question (LIF: 97.3% and ICF: 0). In addition, all respondents indicated that traditional fire use practices continue in the paramo (LIF: 100% and ICF: 0), especially for cattle (*Bos primigenius taurus*) production (LIF: 75.3% and ICF: 0.2 respectively) and crop planting (LIF: 69.3% and IFC: 0.3 respectively). However, when asked if they use the *Minga* (a Kichwa word meaning “collective work”) for planning and executing the burn, 90% (ICF 0.1) agreed that they do not use this community strategy. Likewise, the Saraguro indicated that their burning practices have never gotten out of control in the paramo (LIF: 92% and ICF: 0.1 respectively). Also, when asked if they have noticed high-impact burns in the paramo understood as animal mortality, vegetation loss, and soil degradation, the responses were relatively split between yes and no, with some indicating that yes there have been high-impact burns (LIF: 40.7% and ICF: 0.6) and others indicating that there have been no high impact burns (LIF: 59.3% and ICF: 0.4). Finally, although the Saraguro report that they carry out traditional burns on paramo vegetation, they state that they have not received training from state programs (e.g., the Amazonía sin Fuego “PASF” project), so they continue to feel that there is a lack of concern and implementation of training programs for this important ecosystem (LIF: 94.7% and ICF: 0.1 respectively).

**Table 1:** Main characteristics of the Saraguro people related to the use of fire in the San Lucas paramo

Characteristics/use of fire	LIF (yes)	LIF (no)	ICF (yes)	ICF (no)
Are there paramos on your farm?	97.3	2.7	0	1
Do traditional fire use practices continue?	100	0	0	1
Do you use the paramo for cattle production?	75.3	24.7	0.2	0.8
Do you use the paramo for crop production?	69.3	30.7	0.3	0.7
Is the *minga* used for fire applications in your community?	10	90	0.9	0.1
Has a fire ever gotten out of hand and you have not been able to control the burning?	8	92	0.9	0.1
Has there been a high-impact fire on the paramo?	40.7	59.3	0.6	0.4
Have you received training from any institution on what to do in case of fire in the paramo?	5.3	94.7	1	0.1

*LIF* level of information fidelity, *ICF* informant’s consensus factor, *High impact* refers to direct damage due to animal mortality, vegetation, and soil degradation

On the other hand, in Saraguro village, most people perform traditional burning practices every year (LIF: 74% and ICF: 0.3 respectively) (Table 2). The best time of the year for burning is the period known as “Veranillo del Niño” (VdN) (LIF: 82.0% and ICF: 0.2 respectively). In addition, 82.0% (ICF 0.2) state that 3 to 4 dry and sunny days are needed to burn in the paramo and that 1 day is needed to plan the burning (68.7%, ICF 0.3). Another important aspect is the transmissibility of knowledge; for example, the majority indicate that they learned the need to burn the paramo from their grandparents (62.7%, ICF 0.4) and their parents (36.0%, ICF 0.6). In addition, when asked how many people in the family participate in the burning process, most agree that two or three people go to the paramo to carry out this activity (66.0%, ICF 0.3) and also show some concern that these burns could trigger high impact fires and thus reduce the floristic (74.0%, ICF 0.3) and faunal diversity of the area (26.0%, ICF 0.7). However, when asked their opinion on whether fire benefits the paramo and the soil, most agree (54.7%, ICF 0.5) that fire improves the quality/fertility of the soil, and therefore produces new and better regrowth of plants (17.3%, ICF 0.8). Finally, to understand what motivates the Indigenous people to burn the paramo, the majority say that they do it to generate a new regrowth of plants (to increase food for livestock and wild animals) (74.1%, ICF 0.3). It is worth noting that to the question of whether the motive for burning is to convert paramo vegetation with other grass species and thus expand grazing (land use change), a minority said that this is what motivates them (3.3%, ICF 1).

**Table 2:** Traditional knowledge of the Saraguro Indigenous people, users of the San Lucas paramo ecosystem, regarding the use of fire and application of burns

Topic	Question	Characteristic	LIF	ICF
I. Months of the year to carry out burns and optimal weather conditions	1. If you practice burning on your land, how often do you burn?	Each month	4.0	1
		Every 3 months	4.7	1
		Every 6 months	17.3	0.8
		Each year	74.0	0.3
	2. At what time of the year do you burn1.	At the end of the rainy season	13.3	0.9
		During the drought period	4.0	1
		At the end of the drought period	0.7	1
		In the “Veranillo del Niño” (VdN)	82.0	0.2
	3. Optimal conditions for burning the moor during the year/sunny days.	1–2 days with plenty of sun	18.0	0.8
		3–4 days with plenty of sun	82.0	0.2
	4. How far in advance do you plan a burn	1 day	68.7	0.3
		2 days	24.7	0.8
		> 3 days	6.7	0.9
II. Traditional knowledge about burning	1. From where did you learn that burning must be done to improve the conditions of the paramo?	From their grandparents	62.7	0.4
		From their parents	36.0	0.6
		Shamans	1.3	1
	2. How many people in the family are involved in the burning process?1.1.	One person	28.7	0.7
		Two or three people	66.0	0.3
		> of three persons	5.3	1
	3. What are the main risks when burning?1.1.	That a wildfire starts and the plants of the paramo are lost?	74	0.3
		That there are accidents and deaths of community members?	0.0	1
		Loss of wild animals	26	0.7
	4. Does fire benefit the vegetation and the soil of the paramo? In what way?	Because it improves the regrowth of vegetation and serves to feed animals	17.3	0.8
		Because it attracts the rains	28	0.7
		Because fire improves soil quality/fertility	54.7	0.5
III. Other uses of fire in the paramo	1. What is the reason for choosing the area to be burned?1.1.	Because of proximity?	2.0	1
		To generate new regrowth of medicinal plants?	20.0	0.8
		To generate new regrowth of food plants for livestock and wild animals?	74.7	0.3
		To create a new place to live?	0.0	1
		To change the vegetation of the paramo for another type of pasture and thus expand grazing.	3.3	1

*LIF* level of information fidelity, *ICF* informant’s consensus factor

Finally, there is shared knowledge between women and men concerning the time of burning (EB) and the objective of burning (OB), which is to improve soil fertility with consequent improvement of crop production and natural regeneration of paramo vegetation. Also, there is an agreement between the two genders with respect to the positive benefits of traditional burning (PBTB) to decrease fuel load and with regard to the frequency of burning (FB), which is once a year. However, there are aspects on which there is no agreement between women and men, so future studies are recommended to learn about these discrepancies. The essential aspects refer to the planning of the burn (PB) since there is no consensus on whether it is accomplished in 1 day or 2. Another aspect is how the knowledge was acquired since there is no concordance on whether they received it from their grandparents or their parents, which requires further investigation by evaluating whether people live with their parents or with their grandparents taking into consideration the high migration rate in the area. Finally, other aspects also indicate that there is no concordance among them such as with the age of the burners and the use of the paramo.

### Use of fire calendar, seasons, and application of ignition techniques and patterns

**Fig. 6:**
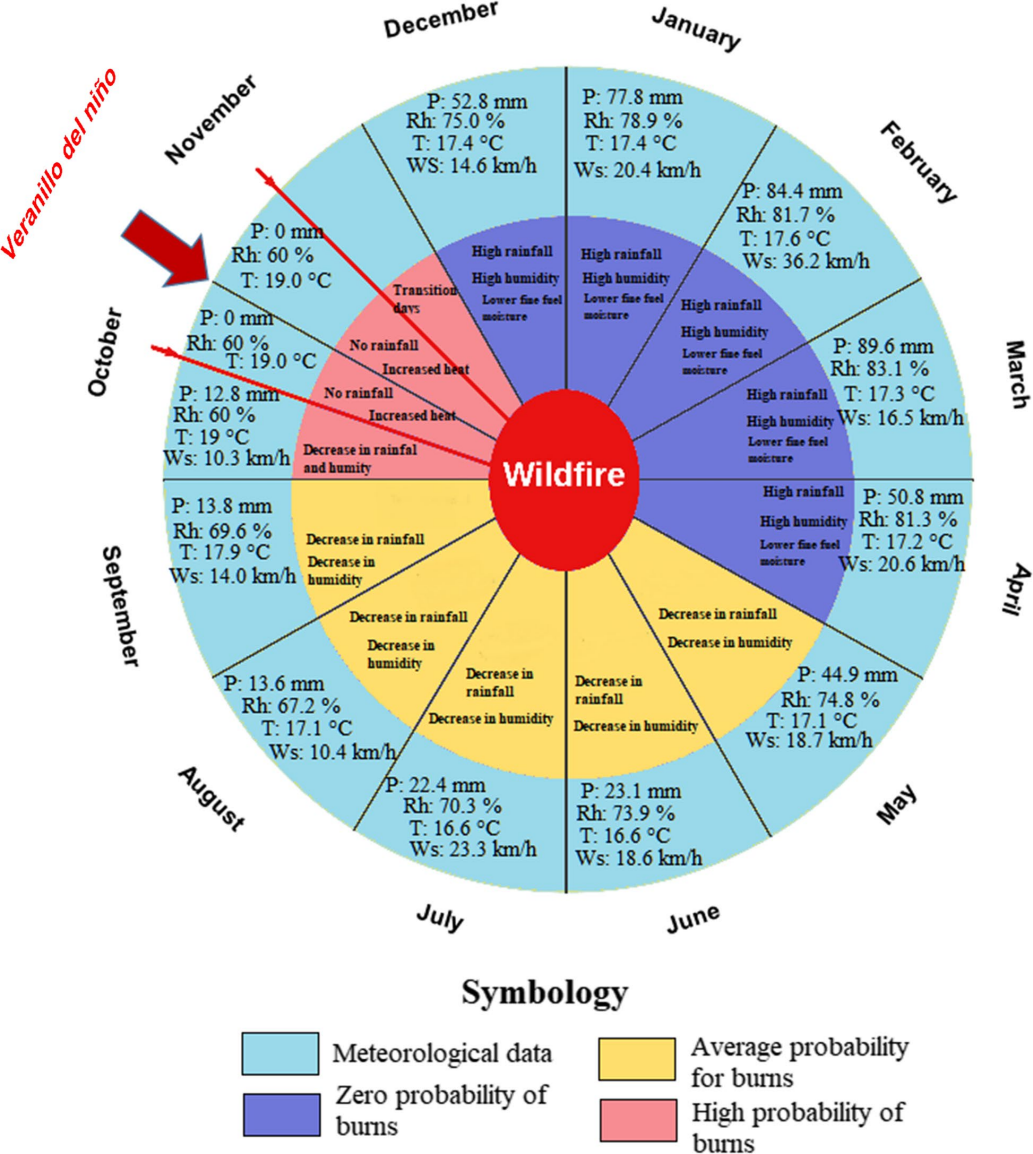
Traditional burning calendar used by the Saraguro Indigenous people, contrasted with NASA meteorological data. Monthly averages of temperature (T), relative humidity (Rh), precipitation (P) (sum), and wind speed (Ws) were used for the four years compared

Figure 6 shows the traditional burning calendar (internal calendar information) compared to NASA weather data (external calendar information). According to Saraguro’s knowledge, there are three climatic phases to take into account for burning. The first phase corresponds to a zero probability, which goes from December to April (blue color) when there is high rainfall and humidity; this coincides with the climatic data where in these months the rainfall reaches 348.9 mm, average relative humidity of 80%, an average temperature of 17.4 °C, and the average wind speed of 21.7 km/h. The second phase corresponds to the months from May to September (yellow color) when precipitation and humidity decrease, and there is an increase in temperature and wind speed. This ancestral knowledge coincides with meteorological data where precipitation decreases to 122.0 mm, relative humidity also decreases to 71.2%, temperature decreases relatively to 17.1 °C, and average wind speed decreases to 17.0 km/h. Under these conditions, the Saraguros need 3 to 4 days without rain and high insolation (solar radiation and low cloud cover) for the vegetation to be ready to be burned (Table 2, topic I, question 3). Finally, the phase of high fire probability corresponds to the VdN phenomenon, which occurs from mid-October to mid-November, which is considered the key time used by the Saraguros to carry out traditional burns. This optimum phase or VdN phenomenon is a very short season, in which there is no rainfall (0 mm), humidity decreases (60%), and temperature increases (19.0 °C), while wind speed remains the same; therefore, the Saraguro consider this phase to be the most effective and have identified this phenomenon as the appropriate season to conduct traditional burns. However, it should be noted that for the Saraguro people, in recent years they have observed that sometimes it occurs earlier or later, but most of the people consulted have stated that the most common period is from mid-October to mid-November.

The ignition techniques and patterns used for traditional burning consist of (i) making a mound of dry grasses (paramo herbaceous vegetation) when weather conditions are optimal and then proceeding to incinerate it simply with the use of matches. And (ii) the ignition pattern consists of making the fuel mound on an almost flat terrain (1–8% slope) in which the wind direction is considered to also know the direction of fire propagation. To prevent the spread of fire to the end of the terrain or to where the spread is to be cut, a clearing strip of approximately 1 m wide is previously constructed with hoes (shovels) until mineral soil material (without vegetation) is obtained. In addition, the generation of possible ignition sparks is controlled by stepping on them or extinguishing them with handmade fireproof covers made of wet grass of the genus *Neurolepis* (Poaceae), which are endemic species of the area. *Neurolepis* is characterized by having a hard stem and leathery leaves. In this way, Saraguro cuts the propagation of the fire.

## Discussion

### Severity of traditional burning

The seasonality of fires that we documented during the 4 years contrasted (Fig. 4) is consistent with other studies in which many fires have been reported to be human-caused (Rother et al. 2020; Carrión-Paladines et al. 2022). However, in our study area due to the lack of information based on historical accounts (Crawford and Brueckheimer 2012) and limited fire records, we estimate that most of these events have an interannual occurrence trend. This annual burning cycle, moreover, is responsible for the occurrence of low-severity fires (Fig. 4) as demonstrated by Sugihara et al. (2006) when describing fire regimes as fuel load reduction tools. Likewise, the results of this study are consistent with that reported by McKemey et al. (2020a, 2020b) who in the savanna of northern Australia reported that there is an annual cycle of fire management by the indigenous Yugul Mangi people. In contrast Ansell and Evans (2019) demonstrated that disruptions to traditional indigenous practices result in extensive and high-severity wildfires (which were not observed in our study area) that occurred predominantly during severe weather conditions, causing significant environmental damage. This is also consistent because most of the territory of the parish of San Lucas is made up of agricultural areas (Fig. 1) and because it is part of the buffer zone of the Corazón de Oro protective forest, where possibly a smaller amount of fuels is found in reference to this protected area. In the case of the paramos, because they are made up of grasses, there is possibly also a low fuel load, which would also produce low-severity fires, as has been reported in recent research (Hutto 2008).

### Traditional use of fire

The indigenous Saraguro continue traditional fire practices as do many Indigenous groups around the world (e.g., Bird et al. 2018; Long et al. 2021). The Saraguro use fire in the parish of San Lucas, where they include it in the management of the paramo ecosystem (Fig. 4, Table 2). This Indigenous group knows through their traditional calendar which months are essential and especially the time of the VdN for burning (Figs. 4 and 6). This coincides with other studies which have observed that Indigenous people from other areas use diverse seasonal calendars according to their cosmovision and ecological parameters based on where they inhabit (McKemey et al. 2021). Regarding the VdN phenomenon, Raffelsbauer et al. (2019) showed that it is characterized by a pronounced low-pressure system east of the Andes that weakens the dominant trade winds. This causes a reduction in cloudiness and the presence of higher outgoing longwave solar irradiance (OLR) under clear skies during the night, and the daily temperature range increases to approximately 25°C, from 2.4°C during the night to 27.1°C during the day (Bendix et al. 2008; Rollenbeck and Bendix 2011a, 2011b). Taking advantage of the VdN phenomenon, and under paramo conditions (Raffelsbauer et al. 2019), the Saraguro produce the low-severity burns (Fig. 4). It is likely that with this type of low-severity burning the Saraguro improve soil chemical conditions (increased pH and organic matter concentration), as recent studies have shown (Pereira et al. 2018), and therefore improve crop yields and the generation of new pasture regrowth for livestock. Furthermore, according to Keeley et al. (2011), these low-severity burns are part of the ecosystem dynamics as it has been shown that many plants have developed germination and zero tolerance mechanisms induced by this type of burning (Richter et al. 2019).

The results of this study are consistent with what occurs with other Indigenous groups around the world. For example, the Tarahumara or Rramuri in Mexico uses fire with the belief that it attracts rain and eliminates weeds to stimulate the growth of grasses (Fulé et al. 2011). Likewise, the Pemon of Venezuela use fire to reduce fuel load, limiting the generation of large fires and also to prepare the soil for crops (shifting agriculture) (Rodríguez 2007). This is corroborated by studies that have shown that this method, known as traditional burning in Ecuadorian forests, generates ecosystem benefits among cultivated areas (Messina and Cochrane 2007; Schritt et al. 2020).

There are also indigenous groups that make traditional use of fire, much like the Saraguro. For example, Anderson (2018) and Long et al. (2021) state that in the western United States, Indigenous communities have long relied on the use of fire to manage their environment and have expressed interest in reasserting their traditional fire management practices (Goode et al. 2018; Clark et al. 2021). In this context, Vázquez-Varela et al. (2022) propose a new paradigm of efficient fire management, which is committed to overcoming strictly biophysical visions of the landscape and claim the need to integrate ancestral knowledge of the communities that inhabit them, generating more efficient proposals for integrated fire management (Higuera et al. 2019). Perhaps, in southern Ecuador, the Indigenous vision of fire should be included to generate new proposals for integrated fire management. This is based on the fact that, in some cases, the Indigenous use of fire produces positive impacts on ecosystems as seems to occur with the Kichwa Saraguro. An example of these benefits is associated with low- and moderate-severity fire regimes, such as those occurring in the study area (Fig. 4). This is confirmed by Bowcutt (2013) and Long and Lake (2018) who demonstrated that many tribes depend on cultural burns and that some Indigenous groups that produce low-severity burns obtain staple foods for the tribes and even for an entire region. Foods such as roots, berries (Lynn et al. 2013), and mushrooms can be promoted by cultural burning as they are exclusively fire-dependent (Pilz et al. 2004; Larson et al. 2016). Conversely, the decline of many of these species has been attributed to fire exclusion (criminalization of fire use), which has facilitated the growth of competing vegetation (Schmidt and Eloy 2020). Therefore, we are convinced that to confirm the benefits of traditional burning by the Kichwa Saraguro, further research is needed to listen to traditional knowledge holders and understand the importance of their long-term observations in their traditional territories. In this way, more elements of judgment will be obtained to opt for the indigenous knowledge of the use of fire as a silvicultural tool. It is also suggested to include soil analysis (physical, chemical, and microbiological properties); the effect on the regeneration of plants, bryophytes, and lichens; and the impacts on migratory fauna to corroborate or refute the convenience or not of incorporating the knowledge of the Indigenous use of fire in the management of the ecosystems of southern Ecuador. But the incorporation of traditional knowledge would be a valuable contribution to clarify this problem.

Finally, in the study area, the analysis of the fire management of both women and men should be expanded, since there are concordances and incongruencies. Therefore, the importance of the role of women should be valued in the study area as it has been shown that women’s participation in traditional uses of fire should be recognized and addressed from a gender approach (Eriksen and Hankins 2015). With the incorporation of women’s knowledge, new information and perspectives on fire use could be found. In this context, it is especially important to assess whether women could be the repositories of data on traditional fire use, as a consequence of the migration of men to urban settings as indicated by Eriksen and Hankins (2014). This constitutes a new research opportunity in southern Ecuador as women in rural areas have been shown to play a key role in decision-making and natural resource management (Aye 2018).

### Legal remedies in Ecuadorian wildfire law compared to other countries

To date, the entire Ecuadorian legal framework does not consider the different types of wildfire severity, in contrast to some South American countries that do. For example, Castillo et al. (2019) report that in Chile they are applying the concept of severity in a new wildfire bill, where they indicate the technical standards by which it will be necessary to perform silvicultural interventions to forests and achieve low-severity fires to reduce dangerous conditions which lead to fire spread. In other regions, such as the USA, they also have similar policies. In the USA, policies recommend the evaluation of low-, mixed, and high-severity fires to improve the performance of ecosystems (Stephens and Ruth 2005). Therefore, fire suppression policies adopted decades ago in Ecuador cause damage to ecosystems, since without knowing the level of fire severity, many ecological formations tend to increase fuels, which increases the risk of large-scale fires, as recent research has shown (Norgaard 2014). Furthermore, given the increased costs of firefighting, the degree of severity should be considered to involve local communities (e.g., Saraguro) as a human resource in firefighting (Everett and Fuller 2011). In this context, the Saraguro can play a key role in fire prevention and response, considering their ancestral knowledge of fire use, as we demonstrate in the low-severity map of this study (Fig. 4). Therefore, based on the results of this study, we recommend including local knowledge of fire use and the concept of fire severity in a new draft law or municipal ordinance for the canton of Loja. In addition, these results can serve to improve the vision of the National Strategy for Integrated Fire Management of Ecuador 2021–2025 that is currently being promoted by the Ministry of Environment, Water and Ecological Transition of Ecuador. In this way, the effectiveness of integrated fire management can be improved and human co-evolution (interculturality) can be considered in the proposals for sustainable ecosystem management. However, it is necessary to conduct more studies with other Indigenous groups in Ecuador, who also live in forest ecosystems different from the area of this study, to obtain more data to generate more effective integrated fire management.

## Conclusions

This study has shown that the traditional use of fire by the Kichwa Saraguro is an important tool for the sustainable management of natural resources in the parish of San Lucas in southern Ecuador. The Saraguro use a traditional burning calendar and carry out these ancestral practices, especially during the phenomenon known as VdN, when weather conditions are optimal. Traditional burns are carried out once a year and produce low-severity fires in cultivated areas, and paramos, producing benefits to ecosystems, as has been pointed out in several studies worldwide. However, we recommend including studies of soil conditions over several years, to verify if the annual frequency of this type of burning is beneficial in the study area. In addition, it is time for Ecuador, and especially in the study area where there is a legal vacuum due to the fear that still exists due to the ancestral use of fire, to start with the formulation of a new legal regulation for the Loja canton, which includes the concepts of Indigenous use of fire and degrees of fire severity. This will serve to contribute with a new management vision and will contribute to improve the National Strategy for Integrated Fire Management in Ecuador 2021–2025, currently promoted by the Ministry of Environment, Water and Ecological Transition. These findings can help decision-makers to design policies, regulations, and proposals for the correct use of fire as a tool for the management of the ecosystems of southern Ecuador affected by wildfires.

## Data Availability

Not applicable.
